# Estimation of Synteny Conservation and Genome Compaction Between Pufferfish (*Fugu*) and Human

**DOI:** 10.1002/(SICI)1097-0061(200004)17:1<22::AID-YEA5>3.0.CO;2-S

**Published:** 2000

**Authors:** Aoife McLysaght, Anton J. Enright, Lucy Skrabanek, Kenneth H. Wolfe

**Affiliations:** 1 Department of Genetics University of Dublin Trinity College Dublin 2 Ireland; 2 Computational Genomics Group Research Programme The European Bioinformatics Institute EMBL Cambridge Outstation Cambridge CB10 1SD UK

## Abstract

Background: Knowledge of the amount of gene order and synteny conservation between two species gives insights to the extent and mechanisms of divergence. The vertebrate *Fugu rubripes* (pufferfish) has a small genome with little repetitive sequence which makes it attractive as a model genome. Genome compaction and synteny conservation between human and *Fugu* were studied using data from public databases.

Methods: Intron length and map positions of human and *Fugu* orthologues were compared to analyse relative genome compaction and synteny conservation respectivley. The divergence of these two genomes by genome rearrangement was simulated and the results were compared to the real data.

Results: Analysis of 199 introns in 22 orthologous genes showed an eight-fold average size reduction in *Fugu*, consistent with the ratio of total genome sizes. There was no consistent pattern relating the size reduction in individual introns or genes to gene base composition in either species. For genes that are neighbours in *Fugu* (genes from the same cosmid or GenBank entry), 40–50% have conserved synteny with a human chromosome. This figure may be underestimated by as much as two-fold, due to problems caused by incomplete human genome sequence data and the existence of dispersed gene families. Some genes that are neighbours in *Fugu* have human orthologues that are several megabases and tens of genes apart. This is probably caused by small inversions or other intrachromosomal rearrangements.

Conclusions: Comparison of observed data to computer simulations suggests that 4000–16 000 chromosomal rearrangements have occured since *Fugu* and human shared a common ancestor, implying a faster rate of rearrangement than seen in human/mouse comparisons.


**Yeast**
*Yeast* 2000; **17**: 22–36.

**Research Article**

### Estimation of synteny conservation and genome compaction between pufferfish (*Fugu*) and human

Aoife McLysaght, Anton J. Enright[†], Lucy Skrabanek and Kenneth H. Wolfe*
*Department of Genetics, University of Dublin, Trinity College, Dublin 2, Ireland*

*Correspondence to:
K. H. Wolfe, Department of Genetics, University of Dublin, Trinity College, Dublin 2, Ireland.
E-mail: khwolfe@tcd.ie

†Current address:
Computational Genomics Group Research Programme, The European Bioinformatics Institute, EMBL Cambridge Outstation, Cambridge CB10 1SD, UK.

Received: 18 January 2000
Accepted: 11 February 2000

### Abstract

**Background:** Knowledge of the amount of gene order and synteny conservation between two species gives insights to the extent and mechanisms of divergence. The vertebrate *Fugu rubripes* (pufferfish) has a small genome with little repetitive sequence which makes it attractive as a model genome. Genome compaction and synteny conservation between human and *Fugu* were studied using data from public databases.

**Methods:** Intron length and map positions of human and *Fugu* orthologues were compared to analyse relative genome compaction and synteny conservation respectively. The divergence of these two genomes by genome rearrangement was simulated and the results were compared to the real data.

**Results:** Analysis of 199 introns in 22 orthologous genes showed an eight-fold average size reduction in *Fugu*, consistent with the ratio of total genome sizes. There was no consistent pattern relating the size reduction in individual introns or genes to gene base composition in either species. For genes that are neighbours in *Fugu* (genes from the same cosmid or GenBank entry), 40–50% have conserved synteny with a human chromosome. This figure may be underestimated by as much as two-fold, due to problems caused by incomplete human genome sequence data and the existence of dispersed gene families. Some genes that are neighbours in *Fugu* have human orthologues that are several megabases and tens of genes apart. This is probably caused by small inversions or other intrachromosomal rearrangements.

**Conclusions:** Comparison of observed data to computer simulations suggests that 4000–16 000 chromosomal rearrangements have occurred since *Fugu* and human shared a common ancestor, implying a faster rate of rearrangement than seen in human/mouse comparisons. Copyright © 2000 John Wiley & Sons, Ltd.

**Keywords:** ***Fugu rubripes*** **(pufferfish); comparative genomics; genome compaction; genome rearrangement; synteny conservation**

### Introduction

Comparative genomics has great potential for maximizing the value of genome sequencing projects. Sydney Brenner and colleagues (Brenner *et al.*, 1993; Elgar *et al.*, 1996) proposed the pufferfish *Fugu rubripes* as a model genome for use in dissecting the human genome. As a vertebrate, *Fugu* is expected to have a similar gene repertoire to human. However, its genome, at ~400 Mb, is approximately 7.5 times smaller than that of human. The reduced amount of repetitive sequence and high gene density make this small genome attractive to molecular biologists.

There are two main factors that will determine whether *Fugu* will be genuinely useful as a model vertebrate. *Fugu* genes must show sufficient similarity to their human orthologues to enable the isolation of a *Fugu* gene with a human (or other mammalian) DNA probe, and vice versa. Further-

Copyright © 2000 John Wiley & Sons, Ltd.

more, knowledge of the extent of linkage conservation between the two genomes will advise as to the feasibility of positional cloning using map information extrapolated from one species to the other (Elgar *et al.*, 1996). Several regions of conserved synteny (but not necessarily conserved gene order) have already been reported between these two genomes (e.g. Baxendale *et al.*, 1995; Trower *et al.*, 1996, Elgar *et al.*, 1999; and references in Table 2).

Exploring the relationship between the human and pufferfish genomes in terms of the extent of synteny conservation and patterns of genome compaction could give insights into the evolution of vertebrate genomes, and could also provide more information on the usefulness of *Fugu* as a model genome. However, at present it is not known how large the syntenic regions are, or how well the gene order is conserved between *Fugu* and human. Recent research on the zebrafish (*Danio rerio*) indicated that for some groups of genes, synteny is conserved in the human but the order of the genes along the syntenic chromosome is different in the two species (Postlethwait *et al.*, 1998). Moreover, many mammalian genes have two zebrafish orthologues, and this is probably due to whole genome or chromosomal duplications that occurred in bony fish (including zebrafish and *Fugu*) after their divergence from the tetrapod lineage (Amores *et al.*, 1998; Gates *et al.*, 1999). It is also not known whether the compaction of the *Fugu* genome relative to the human is uniform throughout the genome, particularly in view of the uneven distribution of genes in the human genome (Ikemura and Wada, 1991; Duret *et al.*, 1995; Deloukas *et al.*, 1998).

Here we have made a comparative genomics study of *Fugu* and the human to investigate the phenomenon of genome compaction and to estimate the level of synteny conservation. There is no genetic map for *Fugu* (it is not possible to breed this fish in the laboratory), so gene linkage is only discernible at the level of genes that were sequenced on the same cosmid or other clone contig. We used two sources of *Fugu* sequence data: large contiguous genomic sequences determined by a variety of laboratories and obtained from GenBank; and 'cosmid skimming' data from the *Fugu* Landmark Mapping Project at the UK MRC HGMP-RC (Elgar, 1996; Elgar *et al.*, 1999). The human map data was obtained from two sources: the Online Mendelian Inheritance in Man database (OMIM 1999); and the physical map of about 30 000 genes (GeneMap '98) constructed from radiation hybrid data by Deloukas *et al.* (1998).

### Materials and methods

### Analysis of homologous introns from *Fugu* and human

The 22 genes included in this analysis were: RPS3, RPS24, DLST, STK9, PAX6, RPS7, APP (low GC3 group); SURF3, SMC1, RPL41, ARF3, CFOS, XLRS1, PCOLCE (medium GC3 group); CSFR1, GH, TSC2, HMOX1, WNT1/INT1, PKD1, G6PD, IT (high GC3 group). All sequences were obtained from GenBank.

### *Fugu* sequence data

SwissProt version 37 (27 July 1999) contains 5406 human proteins. These were compared to the database of *Fugu* 'skimmed' cosmids using TBLASTN (Altschul *et al.*, 1990) using the BLOSUM62 scoring matrix and the SEG filter (Wootton and Federhen, 1996). To remove obvious paralogous hits, only the top hit for each query was retained (provided that it had $P \leq 10^{-15}$) as well as weaker hits that were within a factor of $10^5$ of the top hit. The results of this BLAST search including human map information are available at **http://biotech.bio.tcd.ie/~amclysag/skimmed.html**

A 'skimmed' cosmid was adjudged to contain two genes if two non-overlapping subclones hit different mapped human proteins that are <40% identical in sequence and had $P \leqslant 10^{-15}$ in a BLASTP search. Overlapping *Fugu* cosmids were identified manually and reduced to one entry in Table 1.

*Fugu* proteins from completely sequenced cosmids were compared to the database of human sequences from GeneMap '98 by the TBLASTN programme applying the SEG filter. Only hits with a significance of $\leqslant 10^{-15}$, and that were no more than $10^5$ less likely than the top hit, were accepted. Only the best hit per chromosome was included in further analysis.

Some of the limitations on the analysis of the skimmed cosmids become apparent when the results are compared with the fully sequenced cosmids. Cosmid 168J21 has been fully sequenced under Accession No. AJ010348 (Cottage *et al.*, 1999). The full sequence has three annotated proteins, all of

Copyright © 2000 John Wiley & Sons, Ltd. 

which had human homologues on chromosome 3. In the analysis of the skimmed cosmid sequence only one gene was found. As all three human orthologues are in the SwissProt database, it must be the case that the cosmid subclones do not include the coding sequences of the other two genes.

### Human GeneMap '98 sequences

Deloukas *et al.* (1998) compiled a map (GeneMap '98) of human gene-based markers by radiation hybrid mapping. This includes approximately 30 000 genes. By electronic PCR (Schuler, 1997) they found the corresponding genomic sequence, mRNA and/or EST from the public databases. These results are updated weekly and were downloaded from the NCBI FTP site on 21 December 1998.

A BLAST database of human sequences represented on this radiation hybrid map was created. In order to have comparable map units, only the data from the GeneBridge4 panel (Gyapay *et al.*, 1996) were included. Some parts of the genome are represented more than once in the ePCR output because they have been sequenced more than once as genomic sequence, mRNA and/or EST. Redundancies of this kind were removed, preferentially keeping genomic sequences over mRNA over unfinished sequences over ESTs. The final database had 28 133 entries, totalling 226 506 753 nucleotides.

Some markers in GeneMap '98 are listed with several allocated map positions. In these cases the same position found from several independent experiments or the position with the highest confidence value, as determined by Deloukas *et al.* (1998), was used. Distances within the genome were estimated by counting the number of intervening genes in GeneMap '98. We then adjusted these values for missing data by multiplying this number by 80 000/30 000 (assuming the human genome contains 80 000 genes and the map contains 30 000 genes).

### Computer simulation of genomic rearrangement

In order to make this simulation as realistic as possible, paralogues were assigned at the frequencies observed in the real data. Of the 91 *Fugu* proteins analysed, 78 had hits in the database of mapped human sequences. The distribution of hits is as follows: 47 hit one human sequence, 14 hit two, eight hit three, two hit four, and families of seven, 11, 12, 15, 39, 42, and 59 human proteins were observed once each. More extensive human protein family size data from an intragenome comparison (Imanishi *et al.*, 1997) was used to confirm these results in an independent simulation.

### Results

### Compaction of *Fugu* introns

The *Fugu* genome is much smaller than the human genome, but by virtue of being vertebrate is presumed to have a similar gene repertoire (Brenner *et al.*, 1993). The difference in size must therefore be primarily due to differences in non-coding DNA, including both intergenic and intronic DNA. In vertebrate genomes there is a correlation between gene length and G+C content, with long genes being rare in G+C-rich isochores (Duret *et al.*, 1995). This suggested that there might be a correlation between base composition and the size difference between a human gene and its *Fugu* homologue.

Orthologous *Fugu* and human introns were identified by finding orthologous genomic sequences in GenBank, aligning the protein sequences using the Gap programme (with default settings) of the GCG package, and mapping intron locations onto the protein alignment. Introns were designated orthologous if they were in the same phase and occurred at precisely the same position in the protein alignment produced by Gap. No allowance was made for possible intron sliding during evolution. Using this method, 199 pairs of orthologous introns from 22 genes were found. There were only six cases where we could say with confidence that an intron had been gained or lost after the divergence of these two species. These were all cases where there was an unambiguous alignment of the two protein sequences, and where an intron was present in one sequence but there was no equivalent intron nearby or out of phase in the other organism. Non-coincident introns and introns in ambiguous alignments were excluded from further analysis. Recent research by Hurst *et al.* (1999) tentatively suggests that there may be a dichotomy in the relationship of synonymous GC content and intron size, with warm-blooded vertebrates showing a negative correlation, as previously observed, and cold-blooded vertebrates (including *Fugu*) showing a positive correlation. However, this is not borne out here. In our dataset there is no correlation between intron size and GC3 content of the genes that house them.

Genes were assigned into three equal-sized groups according to their G+C content at codon third positions (GC3) in human, and the lengths of equivalent introns were compared (Figure 1A). The sum of the lengths of all 199 introns in *Fugu* was 59 392 bp, just over eight times smaller than the sum of the lengths of all the human introns (488 726 bp). The large introns of GC3-poor genes are seen to be severely compacted. The compaction averages are 2.9, 6.0 and 14.6, respectively, for the high-, medium-, and low-GC3 groups of genes (Figure 1A), which is broadly consistent with expectations. One-fifth of the *Fugu* introns (41 of the 199) are actually larger than their human counterparts (many only marginally so), and most of these are high-GC3 genes in the human (Figure 1B). However, for the majority of introns (Figure 1B) there does not appear to be any consistent relationship between intron lengths in the two species, or between these and GC3 in their host genes.

The compaction of individual genes, instead of individual introns, was also calculated (Figure 1C, D). Compaction was calculated by dividing the sum of the lengths of introns in a human gene by the sum of the lengths of their *Fugu* orthologues (excluding any non-coincident introns). The compaction values range from 46 (in the APP gene; Villard *et al*., 1998) down to values of less than 1 in two genes (growth hormone and *int1*/*wnt1*), where the *Fugu* gene is larger than the human one. If the GC3 content of a gene and the compaction of its introns are related, then one would expect the greatest compaction to be between human genes with low GC3 and *Fugu* genes with high GC3. Rather surprisingly, there does not appear to be any relationship between the degree of compaction and the base composition in either species (Figure 1C), or the amount of interspecies difference in base composition (Figure 1D). The two most severely compacted genes have similar GC3 content in *Fugu* and human (Figure 1D).

### Synteny conservation between *Fugu* and human

Synteny conservation between two species can be measured in two directions. We can ask, 'What proportion of genes that are syntenic in species A are also syntenic in species B?', or conversely, 'What proportion of genes that are syntenic in B are also syntenic in A?'. These are two distinct quantities, as becomes obvious if one considers a hypothetical case where one of the species has only a single chromosome. The only syntenic genes that are known in *Fugu* are those that have been sequenced on the same clone; there are no large-scale maps of chromosomes. Therefore, we measured *Fugu*/human synteny conservation in terms of the proportion of neighbouring genes (from the same clone or GenBank entry) in *Fugu* that are syntenic in human. We also applied various limits to the physical distance permitted between the syntenic genes in human. Two separate datasets were analysed, as described below.

### Synteny conservation—'cosmid skimming' data

The HGMP-RC *Fugu* landmark mapping project (Elgar, 1996; Elgar *et al*., 1996; Elgar *et al*., 1999) surveyed the *Fugu* genome by limited sequencing ('skimming') of a large number of genomic cosmid clones. Sets of shotgun sequence reads for 850 randomly chosen cosmids are publically available from their website (**http://fugu.hgmp.mrc.ac.uk/**). The data consist of 40 303 sequence reads, with an average of 47 reads per cosmid and 486 bp per read. Each read is assumed to contain no more than one gene.

Because these sequences are short and largely unannotated, we compared them to human data from SwissProt, rather than GeneMap '98 (which contains a large number of EST sequences). Cytogenetic map positions for 3963 of the 5406 human proteins in SwissProt were obtained by following links to OMIM. All 5406 proteins were searched against the *Fugu* cosmid database, using TBLASTN (Altschul *et al*., 1990). Putative orthologous relationships were identified as described in Materials and methods.

A *Fugu* cosmid was considered 'informative' (i.e. it appeared to contain more than one gene, and so contained linkage information) if two different sequence reads hit two different mapped human sequences which did not themselves show significant sequence identity to one another. We identified 48 informative cosmids, containing 58 links between nearby *Fugu* genes (Table 1). For 26 of these links

Copyright © 2000 John Wiley & Sons, Ltd. 

Table 1. *Fugu* ‘skimmed’ cosmids containing homologues of at least two mapped human SwissProt sequences

| Cosmid | Syntenic links[a] + | Syntenic links[a] − | Subclone | SwissProt name[b] | Description | OMIM location |
|---|---|---|---|---|---|---|
| 002I16 | 0 | 1 | bB8 | CGB1 | $G_2$/mitotic-specific cyclin B1 | 5q12 |
| | | | bC1 | UBCG | Ubiquitin-conjugating enzyme E2 G1 | 1q42 |
| 003A22 | 0 | 1 | aD2 | LCFD | Long-chain fatty-acid CoA ligase 4 | Xq22.3 |
| | | | aE9 | AP19 | Clathrin coat assembly protein | Chr.7 |
| 018N05 | 0 | 1 | cB3 | COMT | Catechol *O*-methyltransferase | 22q11.2 |
| | | | cB7 | RYK | Tyr-protein kinase RYK | 3q22 |
| 020M06 | 1 | 1 | bF2 | F16P | Fructose-1-6-bisphosphate | 9q22.2-q22.3 |
| | | | bG9 | GAS1 | Growth arrest specific protein | 9q21.3-q22.1 |
| | | | aE1 | LMG2 | Laminin $\gamma$-2 chain | 1q25-q31 |
| 030J22 | 2 | 0 | aF4 | TRFE | Serotransferrin | 3q21 |
| | | | aF7 | IF4G | Translation initiation factor 4 G | 3q27 |
| | | | aG1 | CLC2 | Chloride channel protein 2 | 3q26-qter |
| 032I12 | 0 | 1 | aD1 | PA2Y | Cytosolic phospholipase A2 | 1q25 |
| | | | aE3 | TSP1 | Thrombospondin 1 | 15q15 |
| 035P08 | 1 | 0 | aC2 | KPT1 | Ser/thr protein kinase PCTAIRE-1 | Xp11.3-p11.23 |
| | | | aD5 | HFC1 | Host cell factor C1 | Xq28 |
| 042H13 | 1 | 2 | aE6 | PIGF | Phosphatidylinositol-glycan synthase F | 2p21-p16 |
| | | | bA4 | GCH1 | GTP cyclohydrolase 1 | 14q22.1-q22.2 |
| | | | bD10 | MSH2 | DNA mismatch repair protein | 2p22-p21 |
| | | | bF8 | CIKA | Voltage gated K channel KV21 | 20q13.2 |
| 050M16 | 1 | 0 | bC5 | CYCH | Cyclin H | 5q13.3-q14 |
| | | | bG2 | GTPA | GTPase-activating protein (GAP) | 5q13.3 |
| 055I13 | 0 | 1 | bD9 | A2MG | $\alpha$-2-Macroglobulin | 12p13.3-p12.3 |
| | | | bE2 | ECH1 | $\delta$3,5-$\delta$2,4-Dienoyl-CoA isomerase | 19q13 |
| 057B20 | 0 | 1 | aC11 | SC14 | sec-14-Like | 17q25.1-q25.2 |
| | | | aH1 | GNT5 | Glucoseaminyltransferase V | 2q21 |
| 059A13 | 0 | 1 | aD6 | VLCS | Very long-chain acyl-CoA synthetase | 15q21.2 |
| | | | aE6 | AMBP | AMBP protein | 9q32-q33 |
| 060I09 | 0 | 1 | aF1 | ITA1 | Integrin $\alpha$-1 | Chr.5 |
| | | | aG3 | ROK | Het. nuclear ribonucleoprotein K | 9q21.32-q21.33 |
| 063J19 | 1 | 1 | aA5 | AGAL | $\alpha$-Galactosidase A | Xq22 |
| | | | aD12 | RL44 | 60S rpL44 | Chr.14 |
| | | | aH4 | DDP | Dystonia protein | Xq22 |
| 068B10 | 1 | 0 | aA9 | MET | Hepatocyte growth factor receptor | 7q31 |
| | | | aC8 | MGR8 | Metabotrophic glutamate receptor 8 | 7q31.3-q32.1 |
| 077E20 | 1 | 1 | bB7 | COGT | Matrix metalloproteinase-14 | 14q11-q12 |
| | | | cC4 | PKD2 | Polycystin 2 | 4q21-q23 |
| | | | cC5 | AF4 | AF-4 protein | 4q21 |
| 081G09 | 1 | 0 | aD12 | CIK4 | Voltage gated K channel protein | 11q13.4-q14.1 |
| | | | aF6 | EAT2 | Excitatory amino acid transporter 2 | 11p13-p12 |
| 082H05 | 0 | 1 | aG5 | KMLS | Myosin light-chain kinase | 3cen-q21 |
| | | | aH4 | NED4 | NEDD-4 protein | 15q |
| 082L03 | 1 | 0 | aD12 | MPCP | Mitochondrial $PO_4$ carrier | 12q23 |
| | | | aF10 | THPA | Thymopoietin-$\alpha$ | 12q22 |
| 086H03 | 1 | 0 | bC4 | DOC2 | Differentially expressed protein 2 | 5p13 |
| | | | cE8 | CO9 | Complement component C9 | 5p13 |
| 096F11 | 0 | 1 | aA7 | WN11 | WNT-11 | 11q13.5 |
| | | | bC7 | ACHD | Acetylcholine receptor $\delta$ chain | 2q33-q34 |
| 103N12 | 1 | 0 | aB9 | RO52 | Ro protein, 52 kDa | 11p15.5 |
| | | | bA6 | COGM | Macrophage meltalloelastase | 11q22.2-q22.3 |
| 104N10 | 1 | 0 | aD3 | FER | FER Tyr protein kinase | 5q21-q22 |
| | | | bA12 | MAN2 | $\alpha$-Mannosidase II | 5q21-q22/20q11.2 |
| 107H09 | 0 | 1 | aF11 | RS12 | 40S rpS12 | 6q |
| | | | aG6 | EYA1 | Eyes absent homologue 1 | 8q13.3 |
| 107N05 | 0 | 2 | aG10 | BCAM | Branched-chain aminotransferase | 19q13 |
| | | | aH4 | GRN | Granulins | Chr.17 |

Copyright © 2000 John Wiley & Sons, Ltd.

Table 1. *Continued*

| | Syntenic links[a] | | | | | |
|---|---|---|---|---|---|---|
| Cosmid | + | − | Subclone | SwissProt name[b] | Description | OMIM location |
| | | | aF6 | EAT2 | Excitatory amino acid receptor 2 | 11p13-p12 |
| 110I12 | 0 | 1 | dA4 | PAK1 | Ser/thr protein kinase PAK-α | 11q13-q14 |
| | | | dD3 | PET1 | Oligopeptide transporter | 13q33-q34 |
| 114M17 | 1 | 0 | bB8 | IHBA | Inhibin β a chain | 7p15-p13 |
| | | | bC3 | EGFR | Epidermal growth factor receptor | 7p12.3-p12.1 |
| 116E05 | 0 | 1 | aB3 | GNT2 | Acetlyglucoseaminyltransferase | 14q21 |
| | | | aE6 | HS9A | Heat shock protein 90-α | 1q21.2-q22 |
| 118A15 | 0 | 1 | cC8 | PERT | Thyroid peroxidase | 2p25 |
| | | | cG3 | VMD2 | Bestrophin | 11q13 |
| 122O20 | 0 | 1 | cA4 | CASR | Extracellular Ca-sensing receptor | 3q13.3-q21 |
| | | | cD1 | CTR2 | Low affinity cationic amino acid transporter | 8p22 |
| 123I02 | 0 | 1 | aC11 | BTG1 | B-cell translocation 1 | 12q22 |
| | | | aE5 | TEF | Thyrotroph embryonic factor | 22q13 |
| 128G19 | 1 | 0 | aC11 | BTG1 | B-cell translocation 1r | 12q22 |
| | | | aE5 | TEF | Thyrotroph embryonic facto | 22q13 |
| 137O18 | 0 | 1 | aE10 | CYA1 | Adenylate cyclase, type I | 7p13-p12 |
| | | | bA4 | BNA1 | Amiloride-sensing brain $Na^+$ channel | 17q11.2-q12 |
| 141H19 | 1 | 0 | aH10 | LDHH | L-Lactate dehydrogenase H chain | 12p12.2-p12.1 |
| | | | aH9 | UGS2 | Glycogen synthetase | 12p12.2 |
| 143P11 | 1 | 0 | aB6 | ANK1 | Ankyrin R | 8p11.2 |
| | | | aD6 | NFM | Neurofilament triplet M protein | 8p21 |
| 145K17 | 0 | 1 | bF3 | RHM1 | Rhombotin-1 | 11p15 |
| | | | cB1 | AHR | AH receptor | 7p15 |
| 147P16 | 2 | 0 | aD1 | DDP | Deafness dystonia protein | Xq22 |
| | | | aF9 | BTK | Tyr-protein kinase BTK | Xq21.3-q22 |
| | | | aG7 | GRA2 | Gly receptor α-2 chain | Xp22.1-p21.2 |
| 155N11 | 1 | 1 | bE7 | SYB2 | Synaptobrevin 2 | 17pter-p12 |
| | | | bH3 | MPP2 | Maguk P55 subfamily member 2 | 17q12-q21 |
| | | | aD7 | UTY | Ubiquitously transcribed TPR on Y | Yq11 |
| 156P04 | 1 | 0 | aH2 | RO52 | Ro protein, 52 kDa | 11p15.5 |
| | | | hC8 | Z195 | Zinc finger protein 195 | 11p15.5 |
| 157C15 | 0 | 1 | aA3 | RIR2 | Ribonucleoside reductase M2 | 2p25-p24 |
| | | | aD10 | RL30 | 60S rpL30 | Chr.8 |
| 159J19 | 1 | 0 | aB1 | MPK4 | MAP kinase kinase 4 | 17p11.2 |
| | | | aD11 | MYSP | Myosin H perinatal skeletal muscle | 17p13.1 |
| 164B03 | 0 | 1 | aD11 | ISL1 | Insulin gene enhancer protein ISL-1 | 5q |
| | | | aD4 | ETFA | Electron transfer flavoprotein-α | 15q23-q25 |
| 165O08 | 0 | 1 | aH7 | UBA1 | Ubiquitin-activating enzyme E1 | Xp11.23 |
| | | | bD10 | DPOE | DNA polymerase ε, subunit A | 12q24.3 |
| 171K15 | 0 | 1 | bB10 | DMK | Myotonin protein kinase | 19q13.2-q13.3 |
| | | | bB6 | BMAL | Brain and muscle ARNT-like 1 | 11p15 |
| 174C18 | 1 | 1 | aD11 | G6PD | G6PD | Xq28 |
| | | | bA1 | CCB3 | $Ca^{2+}$ channel β-3 | 12q13 |
| | | | bB11 | CYA6 | Adenylate cyclase type VI | 12q12-q13 |
| 176J15 | 1 | 0 | aA8 | DESM | Desmin | 2q35 |
| | | | aC5 | PTPN | Protein-tyr phosphatase N | 2q35-q36.1 |
| 192G14 | 1 | 0 | aA2 | ADG | γ-Adaptin | 16q23 |
| | | | aA7 | RFP | Zinc finger protein RFP | Chr.6 |
| 222J11 | 1 | 0 | bE3 | NTTA | Taurine transporter | 3p25-q24 |
| | | | bC4 | ACTQ | $Ca^{2+}$-transporting ATPase | 3p26-p25 |
| Totals: | 26 | 32 | | | | |

[a]The '+' column refers to conserved linkages between *Fugu* and human, and the '−' column refers to non-conserved linkages.
[b]All SwissProt IDs are truncated, omitting '_HUMAN' from each one.

Copyright © 2000 John Wiley & Sons, Ltd. 

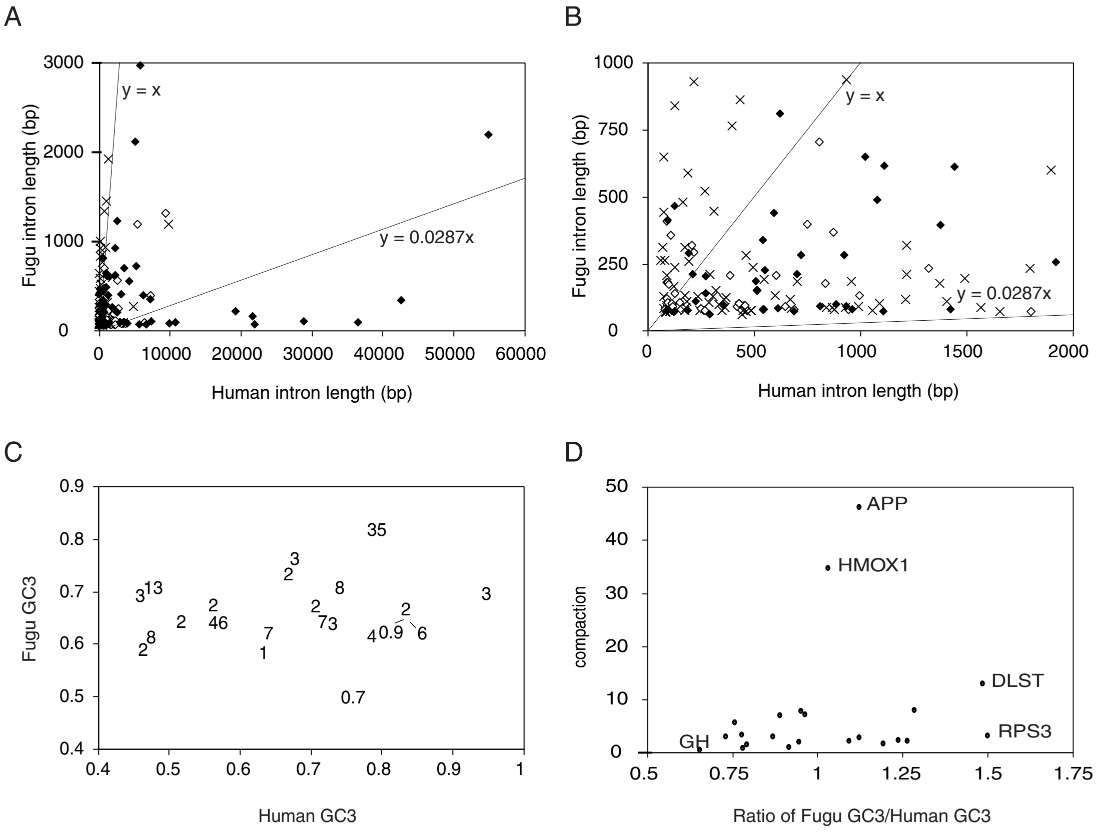

**Figure 1.** (A) Lengths of 199 orthologous introns from *Fugu* and human. The regression line for all data is shown, as is a line of slope 1. The symbols for the points represent different GC3 content categories in the human gene where the black diamond denotes low GC3 (<63.5%), the white diamond denotes medium GC3 (63.5–76%), and the cross denotes high GC3 content (>76%). The categories were designed in such a way as to have equal numbers of genes in each group. The 22 genes from which the introns are derived are named in Materials and methods. (B) Inset of (A) showing only the smaller introns. (C) GC3 content of the 22 orthologous genes whose introns were analysed. The points are replaced by values indicating relative gene compaction. Compaction was calculated by dividing the sum of the lengths of introns of a human gene by the sum of the lengths of their *Fugu* orthologues, ignoring non-conserved introns. (D) Compaction of 22 genes vs. the ratio of GC3 in *Fugu* to that in human. Outlying genes are labelled: APP, amyloid precursor protein; GH, growth hormone; RPS3, ribosomal protein S3; HMOX1, heme oxygenase; DLST, dihydrolipoamide succinyltransferase

(45%), the human homologues are on the same chromosome (i.e. synteny was conserved).

The same *Fugu* Landmark Mapping Project data were recently analysed by Elgar *et al.* (1999). They reported that 'three-quarters' of informative cosmids showed synteny to human. However, it is difficult to account for the differences between our results and theirs as they do not specify what stringency they imposed on the definition of orthology, neither do they indicate which cosmids displayed an orthologous relationship with which human sequences. Perhaps the greatest discrepancy between these analyses is in the number of informative cosmids found (349 by Elgar *et al.* compared to 48 in this study). We expect that this difference is due to a greater stringency employed by us in the designation of orthologues (as described in Materials and methods).

### Synteny conservation—complete *Fugu* genomic sequences

We examined the GenBank annotation of all *Fugu* sequences greater than 5 kb long to look for sequences that coded for two or more proteins. The 21 GenBank entries that fit this criterion

Copyright © 2000 John Wiley & Sons, Ltd. 

Table 2. Details of the completely sequenced *Fugu* cosmids used in this analysis

| Accession No. | Base pairs | Genes included | Reference |
|---|---|---|---|
| af056116 | 148 640 | ACVR1B, ALR, fhh, Ikaros-like, wnt1, wnt10b, ARF3, erbB3, PAS1, rpL41, LRP1 | Gellner and Brenner, 1999 |
| af094327 | 69 056 | SCML2, STK9, XLRS1, PPEF-1, KELCH2, KELCH1, PHKA2, AP19, U2AF1-RS2 | Brunner *et al.*, 1999 |
| u90880 | 61 901 | RNA-H, CAB3B, Adenyl Cyclase-VI, G6PD, LG3P, $Na^+$ channel 2 | Riboldi Tunnicliffe GR *et al.*, unpublished |
| af016494 | 66 729 | GABRB, P55, VAMP-1, PCOLCE, GRMP | Riboldi Tunnicliffe GR *et al.*, unpublished |
| af026198 | 63 155 | L1-CAM, SMC1, CCA1 | Riboldi Tunnicliffe GR *et al.*, unpublished |
| af083221 | 43 373 | Neurotransmitter receptors, YDR140w homologue, glycinamide ribonucleotide transformylase | Reboul *et al.*, 1999 |
| aj010317 | 39 410 | GRM-7, TRIP, Sand, PRGFR3 | Cottage *et al.*, 1999 |
| y15170 | 10 753 | EST00098 homologue, SURF2, SURF4, ASS | Armes *et al.*, 1997 |
| aj010348 | 39 850 | UBE1-like, PRGFR2, calmodulin binding protein kinase | Cottage *et al.*, 1999 |
| al021880 | 37 170 | IGFII, TH, NAP2 | Chen E, *et al.*, unpublished |
| al021531 | 45 565 | WT, Reticulocalbin, PAX6 | Miles *et al.*, 1998 |
| z93780 | 34 807 | CPS3, MLC, MAP2 | Schofield *et al.*, 1997 |
| u92572 | 20 919 | HOXC-9, HOXC-8, HOXC-6 | Aparicio *et al.*, 1997 |
| y15171 | 8 902 | rpL7a (SURF3), SURF1, SURF6 | Armes *et al.*, 1997 |
| af013614 | 55 892 | TSC2, PKD1 | Sandford *et al.*, 1997 |
| af022814 | 37 400 | Zinc finger transcription factor, HMOX1 | Gottgens *et al.*, 1998 |
| af030881 | 5 645 | gag, pol | Poulter and Butler, 1998 |
| aj010316 | 10 959 | Cav-2, Cav-1 | Cottage *et al.*, 1999 |
| u63926 | 23 196 | PDGFR-$\beta$, CSF1R | How *et al.*, 1996 |
| u92573 | 13 583 | HOXA-10, HOXA-9 | Aparicio *et al.*, 1997 |

Cosmids are listed in order of decreasing number of annotated proteins. The list of annotated proteins for each cosmid does not include putative proteins with no known human homologues at the time of submission to the sequence database.

(Table 2) total just under 0.9 Mb and encode 91 annotated proteins (some putative). Genes from the same GenBank entry have a known linkage relationship in the *Fugu* genome because they were sequenced contiguously.

The proteins encoded by these *Fugu* sequences were compared using TBLASTN to the database of human nucleotide sequences whose map positions are known in GeneMap '98 (Deloukas *et al.*, 1998). For some of the *Fugu* sequences, our results confirm previously published analyses (Sandford *et al.*, 1996; Aparicio *et al.*, 1997; Armes *et al.*, 1997; Schofield *et al.*, 1997; Miles *et al.*, 1998; Brunner *et al.*, 1999; Gellner and Brenner, 1999; Reboul *et al.*, 1999).

The results were examined to look for candidate conserved syntenous regions between human and *Fugu*. This was facilitated by a new method for displaying the relative positions of the homologues in the two species. In many cases, such as in the example shown in Figure 2, there was more than one candidate human chromosomal region for conserved synteny. In Figure 2 the *Fugu* sequence (AF056116) appears to have conserved synteny with human chromosome 12 by virtue of having several top scoring BLAST hits to human genes that map close together on that chromosome, largely as described by Gellner and Brenner (1999). What is interesting is that regions on chromosomes 7, 17 and 2 also show synteny with this *Fugu* sequence (including matches to *Fugu* proteins not having homologues on chromosome 12—genes 3, 4, 6, and 14; Figure 2). These are the human chromosomes that contain the HOX clusters and this indicates that the similarity of these human chromosomes to each other extends beyond those clusters, as has been suggested by others (Ruddle *et al.*, 1994).

To examine synteny conservation in a quantitative way, instead of simply the presence or absence of genes on the same chromosome, we calculated the proportion of *Fugu* close neighbours (genes from the same GenBank entry) whose homologues were within a specified distance, $x$, of each other in human. We use the term 'proximity conservation' to denote this property of genes remaining within a specified distance of each other (regardless of gene

Copyright © 2000 John Wiley & Sons, Ltd.

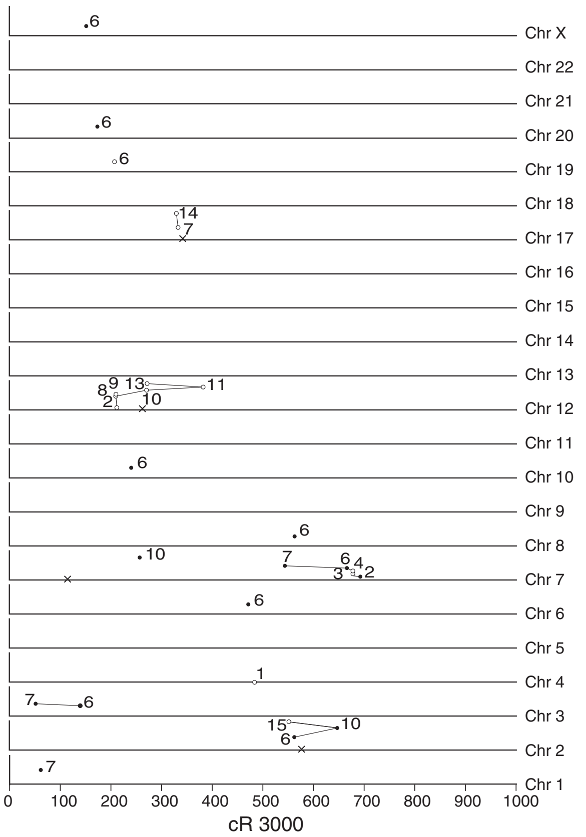

**Figure 2.** Graphical representation of the results of the TBLASTN search of the proteins from *Fugu* sequence AF056116 (Gellner and Brenner, 1999) against a database of mapped human sequences (GeneMap '98). The relative positions of the best hits of each of the 15 annotated *Fugu* proteins from this cosmid are shown for each chromosome in turn. The horizontal axis represents position (measured in centiRads) on the human chromosome in question, and each vertical axis represents the relative order (1–15) of the *Fugu* genes on the *Fugu* cosmid. White dots designate the top-scoring TBLASTN hit for each *Fugu* protein; black dots indicate weaker hits (that are within $10^5$ of the strongest hit). The genes are in the following order in *Fugu*: 1, ACVR1B; 2, ALR; 3, fhh; 4, R05D3.2-like protein; 5, 138E3.2-like protein; 6 Ikaros-like; 7, wnt1; 8, wnt10b; 9, ARF3; 10, erbB3; 11, PAS1; 12, rpl41; 13, 178O23.1-like protein; 14, diaphonous-like protein; 15, LRP1. In addition to the matches shown here (based on data in GeneMap '98), genes 1, 7, 12 and 15 also have homologues on chromosome 12q13 (Kenmochi *et al*., 1998; Gellner and Brenner, 1999). The positions of Hox clusters ABCD are represented by crosses on chromosomes 7, 17, 12 and 2, respectively order). To allow for the uneven distribution of genes in the human genome, the distance $x$ was expressed in terms of the estimated number of intervening genes, instead of in the physical map units (cR) that were used in GeneMap '98 (Deloukas *et al*., 1998). The number of intervening genes was estimated from GeneMap '98 by counting the number of intervening genes appearing on the map between the genes of interest and scaling by a factor of 80 000/30 000 to allow for unsequenced genes. This allows for gene density variation within and between chromosomes. Where more than one human sequence had been assigned to the same map position by Deloukas *et al.* (1998), these sequences were arbitrarily assigned an order.

The results are summarised in Table 3. Only 18% of *Fugu* neighbours have sequenced human homologues that are within 10 genes of one another. This increases to 39% within a limit of 200 intervening genes, and to a maximum of 47% within a limit of 4000 intervening genes (this is effectively no limit, because it is approximately the size of a chromosome). The last value is similar to the synteny estimate from Table 1 (which has no limit on the intervening distance).

### Computer simulation of genomic rearrangement

We used computer simulations to try to relate the observed level of proximity conservation to the number of genomic rearrangements that have occurred since the divergence of *Fugu* and human. The simulation started with a linear array of 80 000 genes, representing the current gene order in *Fugu*. Varying numbers of rearrangements were made in a copy of this genome (representing human) by randomly choosing two endpoints in the genome and inverting the segment in between. To reflect the missing data in the human map, randomly chosen genes were marked 'unmapped' until only 30 000 remained (the number of genes in Deloukas *et al*., 1998). Pairs of genes that are neighbours in *Fugu* were then examined to see if they are neighbours in human, similar to the method of analysis in Tables 1 and 3.

To make the simulation more realistic, we modelled the presence of gene families. Because more than half of all human genes are still not included in the human gene map, there is a real possibility that if the human orthologue of a *Fugu* gene is not mapped, the *Fugu* gene would mistakenly be paired with a mapped human paralogue instead. This could reduce the estimated level of

Table 3. Observed levels of synteny conservation between completely sequenced *Fugu* cosmids and human

| ***Fugu* Accession No.** | **Annotated proteins** | **Proteins with human BLAST hits $P<10^{-15}$** | **Maximum possible links** | **Human chromosome[a]** | **Number of links also present on human chromosome, at different values of *x* intervening genes[b]** | | | | | | |
|---|---|---|---|---|---|---|---|---|---|---|---|
| | | | | | **5** | **10** | **20** | **50** | **200** | **1000** | **4000** |
| AF056116 | 15 | 13 | 12 | 2 | 0 | 1 | 1 | 1 | 1 | 1 | 2 |
| | | | | 3 | 0 | 0 | 0 | 0 | 0 | 1 | 1 |
| | | | | 7 | 0 | 0 | 0 | 1 | 2 | 4 | 5 |
| | | | | 12* | 1 | 1 | 2 | 2 | 3 | 4 | 5 |
| | | | | 17 | 0 | 0 | 0 | 0 | 1 | 1 | 1 |
| AF094327 | 9 | 9 | 8 | 5 | 0 | 0 | 0 | 0 | 0 | 1 | 1 |
| | | | | X* | 1 | 2 | 4 | 4 | 4 | 4 | 4 |
| U90880 | 9 | 6 | 5 | 2 | 0 | 0 | 0 | 0 | 0 | 0 | 1 |
| | | | | 20* | 0 | 0 | 0 | 0 | 1 | 2 | 2 |
| U72484 | 6 | 6 | 5 | 12 | 0 | 0 | 0 | 0 | 2 | 2 | 2 |
| AF016494 | 5 | 4 | 3 | | 0 | 0 | 0 | 0 | 0 | 0 | 0 |
| AF026198 | 5 | 3 | 2 | | 0 | 0 | 0 | 0 | 0 | 0 | 0 |
| AF083221 | 4 | 3 | 2 | | 0 | 0 | 0 | 0 | 0 | 0 | 0 |
| AJ010317 | 4 | 3 | 2 | 3 | 0 | 0 | 0 | 0 | 2 | 2 | 2 |
| Y15170 | 4 | 2 | 1 | 9 | 1 | 1 | 1 | 1 | 1 | 1 | 1 |
| AJ010348 | 3 | 3 | 2 | 3 | 0 | 0 | 0 | 0 | 0 | 0 | 1 |
| AL021880 | 3 | 3 | 2 | 11* | 0 | 0 | 0 | 0 | 1 | 1 | 1 |
| | | | | 12 | 0 | 0 | 0 | 0 | 0 | 0 | 1 |
| AL021531 | 3 | 3 | 2 | 11 | 0 | 0 | 0 | 1 | 1 | 1 | 1 |
| Z93780 | 3 | 3 | 2 | 2 | 0 | 0 | 0 | 1 | 1 | 1 | 1 |
| U92572 | 3 | 3 | 2 | 2 | 0 | 1 | 1 | 1 | 1 | 1 | 1 |
| Y15171 | 3 | 3 | 2 | 9 | 1 | 1 | 2 | 2 | 2 | 2 | 2 |
| AF013614 | 2 | 2 | 1 | 16 | 0 | 0 | 0 | 0 | 0 | 1 | 1 |
| AF030881 | 2 | 1 | 0 | | 0 | 0 | 0 | 0 | 0 | 0 | 0 |
| AF022814 | 2 | 2 | 1 | | 0 | 0 | 0 | 0 | 0 | 0 | 0 |
| AJ010316 | 2 | 2 | 1 | 7 | | 1 | 1 | 1 | 1 | 1 | 1 |
| U63926 | 2 | 2 | 1 | 4 | 0 | 1 | 1 | 1 | 1 | 1 | 1 |
| U92573 | 2 | 2 | 1 | 7 | 1 | 1 | 1 | 1 | 1 | 1 | 1 |
| Totals | | | 57 | | 6 | 9 | 13 | 15 | 22 | 25 | 27 |
| Proximity conservation (%) | | | | | 11 | 16 | 23 | 26 | 39 | 44 | 47 |

[a]In cases where there is more than one candidate human chromosome, * marks the human chromosome with the highest numbers of top scoring BLAST hits, which was used in the calculation of the totals at the bottom. Some of these relationships to human chromosomes have previously been described by the original authors (Sandford *et al.*, 1996; Aparicio *et al.*, 1997; Armes *et al.*, 1997; Schofield *et al.*, 1997; Miles *et al.*, 1998; Brunner *et al.*, 1999; Gellner and Brenner, 1999; Reboul *et al.*, 1999).

[b]The quantity $x$ is the largest allowed distance (in genes) between one of the human homologues and its nearest neighbour in the syntenous group. For the parts of the genome studied here, the intervals of $x=5$, 10, 20, 50, 200, 1000 and 4000 genes correspond to average physical distances of 0.50, 1.27, 2.81, 7.05, 29.14, 144.01 and 494.04 cR, respectively.

Copyright © 2000 John Wiley & Sons, Ltd.

synteny conservation. Simulating this problem requires knowledge of the distribution of gene family sizes, which we addressed in two ways. First, we used the distribution of the numbers of human BLAST hits to the *Fugu* proteins considered in Table 2 (plus annotated putative proteins, totalling 91) as an approximation of the distribution of family sizes. Second, we used the distribution calculated by Imanishi *et al.* (1997) from an all-against-all FASTA comparison of human proteins translated from mapped entries in DDBJ/EMBL/GenBank. In both cases the family sizes were scaled by a factor of 8/3 to account for unsequenced and unmapped genes. The latter (within-genome) method has the advantage that all the hits to a protein represent paralogues, whereas with between-genome comparisons the orthologues must be identified and removed before gene families can be examined. The results from the two methods were similar and only those using the *Fugu* data are presented here.

Paralogous gene families were randomly assigned among the 80 000 genes in the simulated genome, according to the distributions described above. This process resulted in each simulated *Fugu* gene having one human orthologue, and possibly also a list of human paralogues, analogous to a list of BLAST hits. Some of the orthologues and paralogues could be 'unmapped'. Linkage conservation was measured by looking for the human homologues of 1000 pairs of adjacent *Fugu* genes, chosen at random. If the human orthologue of one (or both) of the *Fugu* genes in the pair was 'unmapped', a mapped paralogue from the list was used instead where possible. The extent of linkage conservation in human was then calculated, allowing various intervals between the human homologues. The simulation was run 30 times, looking at 1000 pairs of

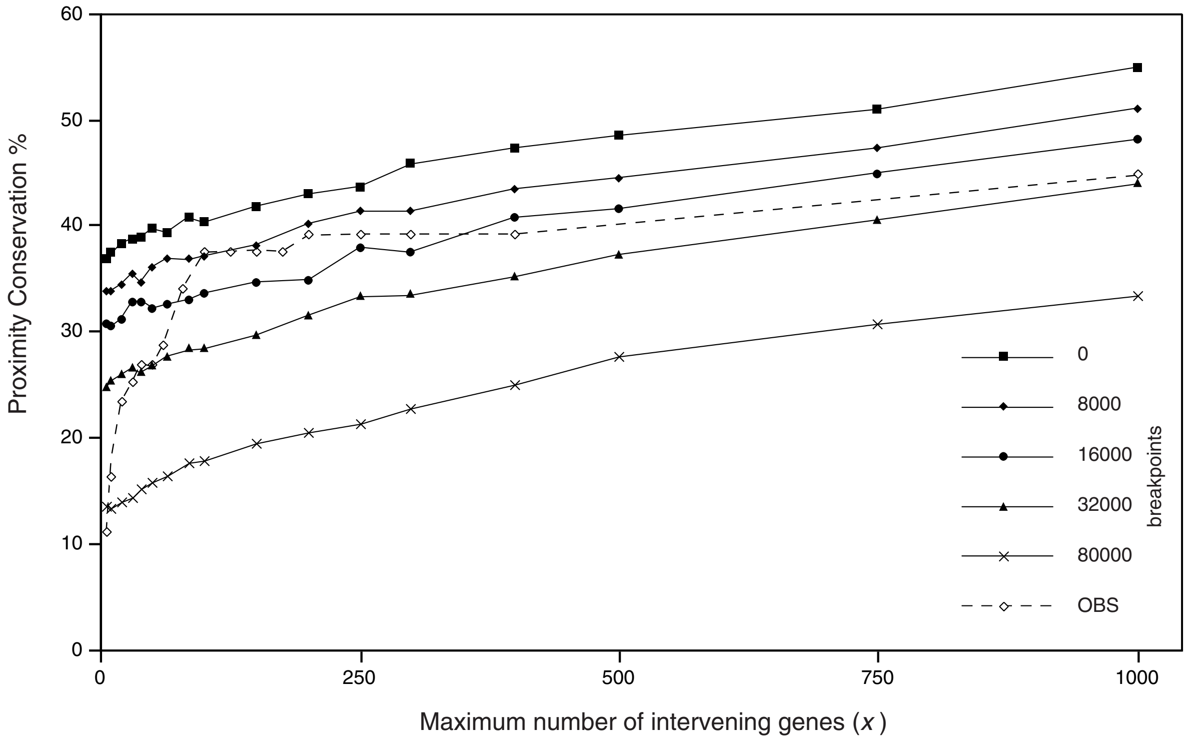

**Figure 3.** Extent of proximity conservation in real and simulated datasets. Proximity was measured allowing different gene distances between the human homologues of pairs of linked *Fugu* genes. The line marked 'OBS' graphs the observed data (Table 3). Average results for 30 computer simulations with 0, 8000, 16 000, 32 000 and 80 000 rearrangement breakpoints are shown (8000 breakpoints = 4000 rearrangements). The *x* axis is the limit used for the distance permitted between two human genes that are homologues of two *Fugu* neighbours, expressed in terms of the estimated number of intervening genes on the chromosome

Copyright © 2000 John Wiley & Sons, Ltd. 

genes each time, with the average results shown in Figure 3.

The most striking feature in the simulation results is that the presence of paralogues in an incompletely-sequenced genome has a substantial effect on the measured extent of linkage conservation. If there have been no genomic rearrangements (top curve in Figure 3), gene order conservation (and thus proximity conservation) should be 100%. However, the measured level is only 37%, because for many gene pairs, one or both of the human orthologues is 'unmapped' and a mapped paralogue at some other location in the genome has been used instead. This makes many linkages appear broken artefactually. Our measures of proximity conservation in the real data may also be underestimated to a similar degree (see Discussion). When the observed data from the fully-sequenced *Fugu* cosmids (Table 3) is plotted on the same axes, its initial slope is much greater than for the simulations (Figure 3). Possible reasons for this are discussed below. At large window sizes the line is approximately the same as the simulations with 8000–32 000 breakpoints.

### Discussion

Although we confirmed the compaction of *Fugu* genes with respect to their human orthologues, we did not observe any strong relationship between gene compaction and the synonymous G+C content of the gene in either species. This may be an artefact of the sample analysed, or it may indicate true randomness in the compaction of the *Fugu* genome. There is an inverse relationship between the average compaction of the genes in each GC3 content category and their average GC3 content, which is consistent with expectations based on the lengths of genes in G+C rich isochores in vertebrate genomes (Duret *et al.*, 1995). However, this relationship is not strong enough to be predictive for individual genes.

The incomplete nature of the human genome data, and the uncertainty regarding whether homologues found in BLAST searches are orthologues or paralogues, reduces our power to examine synteny conservation between *Fugu* and human. The measured proximity conservation depends not only on whether the genes remain close or not, but also on whether they are mapped and sequenced, and if there are paralogues of these genes in the mapped data. The simulations (Figure 3) suggest that the combination of incomplete sequence sets and the presence of gene families may cause the level of proximity conservation to be underestimated substantially, perhaps two-fold.

There is an obvious discrepancy between the slope of the graph of proximity conservation in real data from fully sequenced cosmids, as compared to the results from computer simulations (Figure 3). The observed proximity conservation rises steeply to 37% at a window size of 100 intervening genes, and then plateaus to a shape more like the simulated data. This suggests that the assumptions underlying the simulation are incorrect in some way.

The steep rise may be attributable to three primary factors. One possibility is that the real data is not a random sample of genes from the two organisms. A bias may result from *Fugu*'s role as a model vertebrate genome, inevitably influencing the selection of cosmids for complete sequencing. Cosmids with hypothesised synteny conservation with mammalian genomes may have been chosen preferentially. At least five of the *Fugu* complete sequences used had known synteny conservation with human chromosomes prior to sequencing (Aparicio *et al.*, 1997; Armes *et al.*, 1997; Sandford *et al.*, 1997).

Second, lack of resolution and incomplete data in GeneMap '98 data may affect the results. The arbitrary ordering of human genes that lie in the same radiation hybrid map interval could inflate apparent distances in human, although this effect is unlikely to be significant because the average number of genes per interval in the GeneMap '98 data used here is only 1.98. At least one distance in Table 3 has been overestimated due to missing data in GeneMap '98. This occurs with the genes TSC2 and PKD1 (*Fugu* Accession No. AF013614), which are neighbours in both species (Sandford *et al.*, 1996). However, PKD1 is not present in the map and instead our method identified a PKD1-like sequence elsewhere on chromosome 16 (Loftus *et al.*, 1999).

A third factor may be that our model of rearrangements is too simple. Our model assumed a random distribution of breakpoints throughout the genome, but comparative analysis of the human and mouse maps has shown that, although inter-chromosomal rearrangements seem to have random

Copyright © 2000 John Wiley & Sons, Ltd.

endpoints, the number of intrachromosomal rearrangements is more than expected at random (Ehrlich *et al.*, 1997; Nadeau and Sankoff, 1998). The steep incline at the beginning of the graph may indicate a high frequency of small inversions or other small intrachromosomal rearrangements. Inversions of small segments of chromosome would disrupt gene adjacencies while preserving gene vicinities. This has been proposed by Gilley and Fried (1999), who noticed that some genes that are adjacent in *Fugu* are 2–4 Mb apart in human. Further examples from our study include wnt10b, ARF3 and erbB3. These genes are adjacent in *Fugu* (Gellner and Brenner, 1999). In human, wnt10b and ARF3 are adjacent but erbB3 is separated from them by an estimated distance of 603 genes (226 mapped GenBank sequences scaled by 8/3 to allow for missing data) or 7.5 Mb [estimated from the map distance of 31 cR; chromosome 12 has an average of 234 kb/cR (Gyapay *et al.*, 1996)].

It is likely that the initial portions of the simulations in Figure 3 are not directly comparable with the observed data. However, as the window size gets larger the graph lines are approximately parallel to the plot of the observed data. From these an estimate of the extent of rearrangement since the divergence of these two lineages 400 million years ago is 8000–32 000 breakpoints (i.e. 4000–16 000 reciprocal translocations or inversions). This is higher than expected from comparisons of the human and mouse genomes, which diverged 100 million years ago and have only had an estimated 180 rearrangements (Nadeau and Taylor, 1984; Nadeau and Sankoff, 1998). Adjusting our simulations to incorporate a bias towards small rearrangements would only increase the estimated number of rearrangements since the *Fugu*–human divergence, making the discrepancy in rates even greater. Our estimate of the number of breakpoints depends somewhat on the estimated number of genes in the genome. Simulations based on a gene number of 61 000 instead of 80 000 (see Dunham *et al.*, 1999) led to an estimate of roughly 6000–12 000 breakpoints. Simulations using a gene number of 143 000 (recently suggested by Incyte Pharmaceuticals; see Dickson, 1999) produced the unexpected result that that no rearrangements were called for: the number of proximities observed in *Fugu* exceeded what would be expected due to the now-sparse sampling of genes, so either Incyte's estimate is unrealistic or some of the orthologues listed in Table 3 are actually paralogues.

Another possible shortcoming of our analysis is the presence of short ESTs (which are not necessarily coding sequence) in the human DNA database used here, resulting in an overestimate of the frequency with which we can expect to find orthologues in this dataset from an amino acid level search. However, this is unlikely to have a great effect on the results, because we found that 78% of a random sample of over 500 human proteins submitted to TREMBL after we downloaded GeneMap '98 were represented in the database. The gene family data is also likely to be oversimplified, as it is based on results from only 91 *Fugu* proteins. The Imanishi *et al.* (1997) data is from a larger set of proteins but is not as easy to relate to the human dataset used in this analysis.

Because we have approached the question of synteny conservation from the perspective of known gene adjacencies in *Fugu*, the proposed genome duplication in the bony fish lineage (Amores *et al.*, 1998; Meyer and Schartl, 1999), followed by differential gene loss, should not influence the results. If genes in the ancestral genome were ordered ABCD and this was duplicated in the fish lineage, differential gene loss could result in paralogous chromosomes, one bearing AC and another bearing BD. If synteny of these genes had not been disturbed, then the human genome would still contain the four genes arranged ABCD. If we were counting conservation of human linkages in *Fugu*, then we might plausibly have selected genes A and B for analysis and found that they are not syntenous in *Fugu*, an artefact of gene loss rather than genome rearrangement. However, as we are starting from the complementary viewpoint (given known relationships in *Fugu*), the only possible questions are 'Are A and C syntenous in the human genome?', and 'Are B and D syntenous in the human genome?', which is true in both cases. It is, however, possible that differential gene loss (after the genome duplication) in the *Fugu* lineage has contributed to the reduction of some intergenic distances as compared to human (e.g. the distance from A to C in the hypothetical example). This may also contribute to the steep initial slope seen in Figure 3. One example of apparent differential gene loss may already have been discovered in the case of the genes IGF2 and TH (insulin-like growth factor and tyrosine hydroxylase), which are adjacent in

Copyright © 2000 John Wiley & Sons, Ltd. 

*Fugu* but separated by one intervening gene (insulin) in human (E. Chen *et al.*, unpublished; GenBank Accession No. AL021880; Lucassen *et al.*, 1993). Patterns of gene loss and gene order evolution should become clearer when more long homologous sequences from these species become available.

### Acknowledgements

We thank Cathal Seoighe for helpful discussions and a critical appraisal of this manuscript, and Andrew Lloyd for useful suggestions. Supported by the Health Research Board (Ireland).

### References

Altschul SF, Gish W, Miller W, Myers EW, Lipman DJ. 1990. Basic local alignment search tool. *J Mol Biol* **215**: 403–410.

Amores A, Force A, Yan Y, Joly L, Amemiya C, Fritz A, Ho RK, Langeland J, Prince V, Wang YL, Westerfield M, Ekker M, Postlethwait JH. 1998. Zebrafish *Hox* clusters and vertebrate genome evolution. *Science* **282**: 1711–1714.

Aparicio S, Hawker K, Cottage A, *et al.* 1997. Organization of the *Fugu rubripes Hox* clusters: evidence for continuing evolution of vertebrate *Hox* complexes. *Nature Genet* **16**: 79–83.

Armes N, Gilley J, Fried M. 1997. The comparative genomic structure and sequence of the *surfeit* gene homologs in the puffer fish *Fugu rubripes* and their association with CpG-rich islands. *Genome Res* **7**: 1138–1152.

Baxendale S, Abdulla S, Elgar G, *et al.* 1995. Comparative sequence analysis of the human and pufferfish Huntington's disease genes. *Nature Genet* **10**: 67–76.

Brenner S, Elgar G, Sandford R, Macrae A, Venkatesh B, Aparicio S. 1993. Characterisation of the pufferfish (*Fugu*) genome as a compact model vertebrate genome. *Nature* **366**: 265–268.

Brunner B, Todt T, Lenzner S, *et al.* 1999. Genomic structure and comparative analysis of nine *Fugu* genes: conservation of synteny with human chromosome Xp22.2-p22.1. *Genome Res* **9**: 437–448.

Cottage A, Clark M, Hawker K, *et al.* 1999. Three receptor genes for plasminogen related growth factors in the genome of the puffer fish *Fugu rubripes*. *FEBS Lett* **443**: 370–374.

Deloukas P, Schuler GD, Gyapay G, *et al.* 1998. A physical map of 30 000 human genes. *Science* **282**: 744–746.

Dickson D. 1999. Gene estimate rises as US and UK discuss freedom of access. *Nature* **401**: 311

Dunham I, Shimizu N, Roe BA, *et al.* 1999. The DNA sequence of human chromosome 22. *Nature* **402**: 489–495.

Duret L, Mouchiroud D, Gautier C. 1995. Statistical analysis of vertebrate sequences reveals that long genes are scarce in GC-rich isochores. *J Mol Evol* **40**: 308–317.

Ehrlich J, Sankoff D, Nadeau JH. 1997. Synteny conservation and chromosome rearrangements during mammalian evolution. *Genetics* **147**: 289–296.

Elgar G. 1996. Quality not quantity: the pufferfish genome. *Hum Mol Genet* **5**: 1437–1442.

Elgar G, Clark MS, Meek S, *et al.* 1999. Generation and analysis of 25 Mb of genomic DNA from the pufferfish *Fugu rubripes* by sequence scanning. *Genome Res* **9**: 960–971.

Elgar G, Sandford R, Aparicio S, Macrae A, Venkatesh B, Brenner S. 1996. Small is beautiful: comparative genomics with the pufferfish (*Fugu rubripes*). *Trends Genet* **12**: 145–150.

Gates MA, Kim L, Egan ES, *et al.* 1999. A genetic linkage map for zebrafish: comparative analysis and localization of genes and expressed sequences. *Genome Res* **9**: 334–347.

Gellner K, Brenner S. 1999. Analysis of 148 kb of genomic DNA around the *wnt1* locus of *Fugu rubripes*. *Genome Res* **9**: 251–258.

Gilley J, Fried M. 1999. Extensive gene order differences within regions of conserved synteny between the *Fugu* and human genomes: implications for chromosomal evolution and the cloning of disease genes. *Hum Mol Genet* **8**: 1313–1320.

Gottgens B, Gilbert JGR, Barton LM, *et al.* 1998. The pufferfish *SLP-1* gene, a new member of the *SCL/TAL-1* family of transcription factors. *Genomics* **48**: 52–62.

Gyapay G, Schmitt K, Fizames C, *et al.* 1996. A radiation hybrid map of the human genome. *Hum Mol Genet* **5**: 339–346.

How GF, Venkatesh B, Brenner S. 1996. Conserved linkage between the puffer fish (*Fugu rubripes*) and human genes for platelet-derived growth factor receptor and macrophage colony-stimulating factor receptor. *Genome Res* **6**: 1185–1191.

Hurst LD, Brunton CF, Smith NG. 1999. Small introns tend to occur in GC-rich regions in some but not all vertebrates. *Trends Genet* **15**: 437–439.

Ikemura T, Wada K. 1991. Evident diversity of codon usage patterns of human genes with respect to chromosome banding patterns and chromosome numbers; relation between nucleotide sequence data and cytogenetic data. *Nucleic Acids Res* **19**: 4333–4339.

Imanishi T, Endo T, Gojobori T. 1997. An exhaustive search for extensive chromosomal regions duplicated within the human genome. HGM '97 Poster (March 1997, Toronto, Canada). **http://www.cib.nig.ac.jp/dda/timanish/dup.html**

Kenmochi N, Kawaguchi T, Rozen S, *et al.* 1998. A map of 75 human ribosomal protein genes. *Genome Res* **8**: 509–523.

Loftus BJ, Kim UJ, Sneddon VP, *et al.* 1999. Genome duplications and other features in 12 Mb of DNA sequence from human chromosome 16p and 16q. *Genomics* **60**: 295–308.

Lucassen AM, Julier C, Beressi JP, *et al.* 1993. Susceptibility to insulin dependent diabetes mellitus maps to a 4.1 kb segment of DNA spanning the insulin gene and associated VNTR. *Nature Genet* **4**: 305–310.

Meyer A, Schartl M. 1999. Gene and genome duplications in vertebrates: the one-to-four (-to-eight in fish) rule and the evolution of novel gene functions. *Curr Opin Cell Biol* **11**: 699–704.

Miles C, Elgar G, Coles E, Kleinjan D, van Heyningen V, Hastie N. 1998. Complete sequencing of the *Fugu WAGR* region from *WT1* to *PAX6*: dramatic compaction and conservation of synteny with human chromosome 11p13. *Proc Natl Acad Sci U S A* **95**: 13068–13072.

Nadeau JH, Sankoff D. 1998. Counting on comparative maps. *Trends Genet* **14**: 495–501.

Copyright © 2000 John Wiley & Sons, Ltd. 

Nadeau JH, Taylor BA. 1984. Lengths of chromosomal segments conserved since divergence of man and mouse. *Proc Natl Acad Sci U S A* **81**: 814–818.

OMIM. 1999. Online Mendelian Inheritance in Man, OMIM (TM). Center for Medical Genetics, Johns Hopkins University, Baltimore, MD, and National Center for Biotechnology Information, National Library of Medicine, Bethesda, MD.

Postlethwait JH, Yan YL, Gates MA, *et al.* 1998. Vertebrate genome evolution and the zebrafish gene map. *Nature Genet* **18**: 345–349.

Poulter RTM, Butler MI. 1998. A retrotransposon family from the pufferfish (fugu) *Fugu rubripes*. *Gene* **215**: 241–249.

Reboul J, Gardiner K, Monneron D, Uze G, Lutfalla G. 1999. Comparative genomic analysis of the *interferon/interleukin-10* receptor gene cluster. *Genome Res* **9**: 242–250.

Ruddle FH, Bentley KL, Murtha MT, Risch N. 1994. Gene loss and gain in the evolution of the vertebrates. *Dev Suppl* 155–161.

Sandford R, Sgotto B, Aparicio S, *et al.* 1997. Comparative analysis of the polycystic kidney disease 1 (*PKD1*) gene reveals an integral membrane glycoprotein with multiple evolutionary conserved domains. *Hum Mol Genet* **6**: 1483–1489.

Sandford R, Sgotto B, Burin T, Brenner S. 1996. The tuberin (*TSC2*), autosomal dominant polycystic kidney disease (*PKD1*), and somatostatin type V receptor (*SSTR5*) genes form a synteny group in the *Fugu* genome. *Genomics* **38**: 84–86.

Schofield JP, Elgar G, Greystrong J, *et al.* 1997. Regions of human chromosome 2 (2q32-q35) and mouse chromosome 1 show synteny with the pufferfish genome (*Fugu rubripes*). *Genomics* **45**: 158–167.

Schuler GD. 1997. Sequence mapping by electronic PCR. *Genome Res* **7**: 541–550.

Trower MK, Orton SM, Purvis IJ, *et al.* 1996. Conservation of synteny between the genome of the pufferfish (*Fugu rubripes*) and the region on human chromosome 14 (14q24.3) associated with familial Alzheimer disease (*AD3* locus). *Proc Natl Acad Sci U S A* **93**: 1366

Villard L, Tassone F, Crnogorac-Jurcevic T, Clancy K, Gardiner K. 1998. Analysis of pufferfish homologues of the AT-rich human *APP* gene. *Gene* **210**: 17–24.

Wootton JC, Federhen S. 1996. Analysis of compositionally biased regions in sequence databases. *Methods Enzymol* **266**: 554–571.

Copyright © 2000 John Wiley & Sons, Ltd. 
